# Editorial: AI and inverse methods for building digital twins in neuroscience

**DOI:** 10.3389/fncom.2025.1684335

**Published:** 2025-09-08

**Authors:** Alain Nogaret, Ana Mirallave-Pescador, Maik Kschischo

**Affiliations:** ^1^Department of Physics, University of Bath, Bath, United Kingdom; ^2^Department of Neurosurgery, King's College Hospital, Foundation Trust, London, United Kingdom; ^3^Department of Neurophysiology, King's College Hospital, Foundation Trust, London, United Kingdom; ^4^Faculty of Computer Science, Universität Koblenz, Koblenz, Germany

**Keywords:** deep learning–artificial intelligence, neuroscience, convolution neural networks (CNN), recurrent neural networks (RNN), data assimilation (DA)

Deep learning is revolutionizing Neuroscience and Healthcare. One driver for this change is the exponential growth in the volume of biomedical data generated by modern medical imaging technology and automated data acquisition systems, such as automated patch clamps. Another driver is the increasing reliance of clinical diagnosis on deep learning algorithms to detect abnormalities in MRI scans. By automating the analysis of MRI images, algorithms free clinical staff from repetitive and time-consuming tasks while removing subjectivity in the diagnosis and classification of brain tumors. Deep learning algorithms routinely assist neurosurgeons during critical operations, for example by stimulating surrounding brain tissue during tumor resection. Deep learning algorithms are also increasingly capable of forecasting epileptic seizures and assisting researchers in understanding how language is coded in the brain. Digital twins of brain activity trained on electroencephalographic time series have predicted epileptic seizures and connectivity changes in the brain during language comprehension. Machine learning is also progressing neuroscience by modelling biocircuits at the single neuron level. Recursive neural networks trained on electrophysiological data are making accurate predictions of the voltage oscillations of central pattern generators. The dynamics of individual ionic currents is also inferred to a good degree of accuracy when additional information in the form of surrogate model is provided. This importantly suggests that the dynamics of the membrane voltage which is observed and the ionic current waveforms which cannot be directly measured may be reconstructed from the analysis of electrophysiological time series.

The deep learning techniques described in this collection fall into three classes. Convolution neural networks are used to analyze medical images where filters associated with biomarkers may be inserted between neural layers to extract and classify features of interest. In contrast, recurrent neural networks keep the memory of past inputs allowing patterns to be identified in sequences of data such as electrophysiological recordings or electroencephalograms. The third class of methods rely on supervised deep learning to estimate model parameters from experimental time series. Here the algorithm is provided with the equations of the dynamical system. Once training is complete, the optimized model predicts the waveforms of both observed and unobserved state variables within and beyond the training window.

The article by Kujawa et al. demonstrates the power of automatic segmentation within a convolution neuronal network to identify and classify tumors of the auditory canal. Findings over a cohort of patients show that deep learning achieves sufficient accuracy to replace human expert diagnosis except for the smallest tumors. The article by Acker et al. builds a map of brain connectivity between specific brain regions that are activated by auditory stimulation. The brain regions are encoded as the nodes of graph neural network and the connectivity between brain regions as the lines of the graph. They successfully train this network to recognize and then predict changes in functional brain connectivity during the comprehension of a spoken language. The article by Mallick and Baths develops a deep learning framework to detect epileptic seizures by identifying precursor markers from EEG images. Recurrent neural networks are used to tag features associated with seizures in large datasets in combination with a convolution neural network to categorize them. This approach achieved high recognition accuracy offering a potential tool for the diagnosis of epilepsy. The article by Burghi et al. shows how recurrent neural networks can be trained to predict the electrical activity of crustacean central pattern generators. They augment the predictive power of their recurrent network by introducing teacher forcing and multiple shooting to successfully predict the dynamics of the half center oscillator. The article by Wells et al. synchronizes a conductance model to electrophysiological recordings of hippocampal neurons to predict membrane voltage oscillations and infer the current waveforms of all ion channel on a neuron in one go. They performed pharmacological manipulations blocking specific ion channels with well-known antagonists and carefully calibrated dosing to show that that supervised deep learning correctly predicts which ion channel has been blocked and by how much.

